# Concomitant post-traumatic ocular and cerebral fat embolism syndrome and thrombotic pulmonary embolism: A case report

**DOI:** 10.1097/MD.0000000000029331

**Published:** 2022-06-17

**Authors:** Ying-Sheng Li, Yi-Hsueh Liu, Hung-Da Chou, Hsiang-Jui Tseng, Yin-Chih Fu, Wen-Chih Liu

**Affiliations:** a Department of Neurology, Kaohsiung Medical University Hospital, Kaohsiung Medical; b University, Kaohsiung, Taiwan; b Department of Internal Medicine, Kaohsiung Municipal Siaogang Hospital, Kaohsiung Medical University, Kaohsiung, Taiwan; c Department of Ophthalmology, Chang Gung Memorial Hospital, Linkou Medical Center, Taoyuan, Taiwan; d College of Medicine, Chang Gung University, Taoyuan, Taiwan; e Department of Orthopedic Surgery, Kaohsiung Medical University Hospital, Kaohsiung, Taiwan; f Graduate Institute of Medicine, Kaohsiung Medical University, Kaohsiung, Taiwan; g Department of Orthopedic Surgery, Kaohsiung Municipal Ta-Tung Hospital, Kaohsiung, Taiwan; h Ph.D Program in Biomedical Engineering, College of Medicine, Kaohsiung Medical University, Kaohsiung, Taiwan.

**Keywords:** fat embolism syndrome, femur fracture, hip fracture, non-vitamin K oral anticoagulant, pulmonary embolism, retinal artery occlusion, visual field defect

## Abstract

**Rationale:**

Fat embolism syndrome (FES) is composed of a triad of symptoms, including respiratory distress, neurologic deficit, and petechiae. Respiratory distress usually presents first before the other symptoms. Thrombotic pulmonary embolism (TPE) is a differential diagnosis of FES. Trauma is a risk factor for both diseases; however, co-occurrence is rare.

**Patient concerns:**

A 35-year-old male patient presented with altered consciousness, focal neurologic deficit, and respiratory distress after a left femoral subtrochanteric fracture and subsequent open reduction and internal fixation with an intramedullary nail.

**Diagnosis:**

Computed tomography pulmonary angiography (CTPA) revealed lower pulmonary artery filling defects and ground-glass opacities in bilateral lung, indicating TPE and FES, respectively.

**Interventions:**

Heparin was initially added and subsequently switched to apixaban. The symptoms improved quickly without major bleeding complications.

**Lession Subsections:**

Concomitant TPE and FES after trauma are rare and require different treatment approaches. Due to clinical similarities, prompt chest CTPA was advised to detect TPE that was treated with anticoagulant therapy instead of supportive care for FES.

## 1 Introduction

Fat embolism syndrome (FES) is a rare condition that presents with respiratory distress, neurologic deficits, and petechiae. It usually occurs following long bone fractures with an onset after 24–72 hours.^[1]^ Respiratory distress is the most common and earliest symptom of FES. The treatment for FES comprises supportive care, and the mortality rate is 1.2%.^[2]^ Hence, supportive care is sufficient, and anticoagulants currently play no role in the treatment strategy.^[2,3]^

Thrombotic pulmonary embolism (TPE) is caused by pulmonary artery obstruction by the thrombus. The variety of clinical presentations can help differentiating TPE from respiratory distress. However, trauma can also cause acute pulmonary embolism.^[4]^ The onset of FES and TPE have similar clinical presentations. However, their treatments are different. Empirical anticoagulants are advised for TPE except in patients with high risk for bleeding.^[5]^ Early diagnosis is important to determine the therapy. Here, we present a rare case of concomitant FES and PE that was treated with a non-vitamin K oral anticoagulant (NOAC).

## 2 Case report

**Figure 1:**
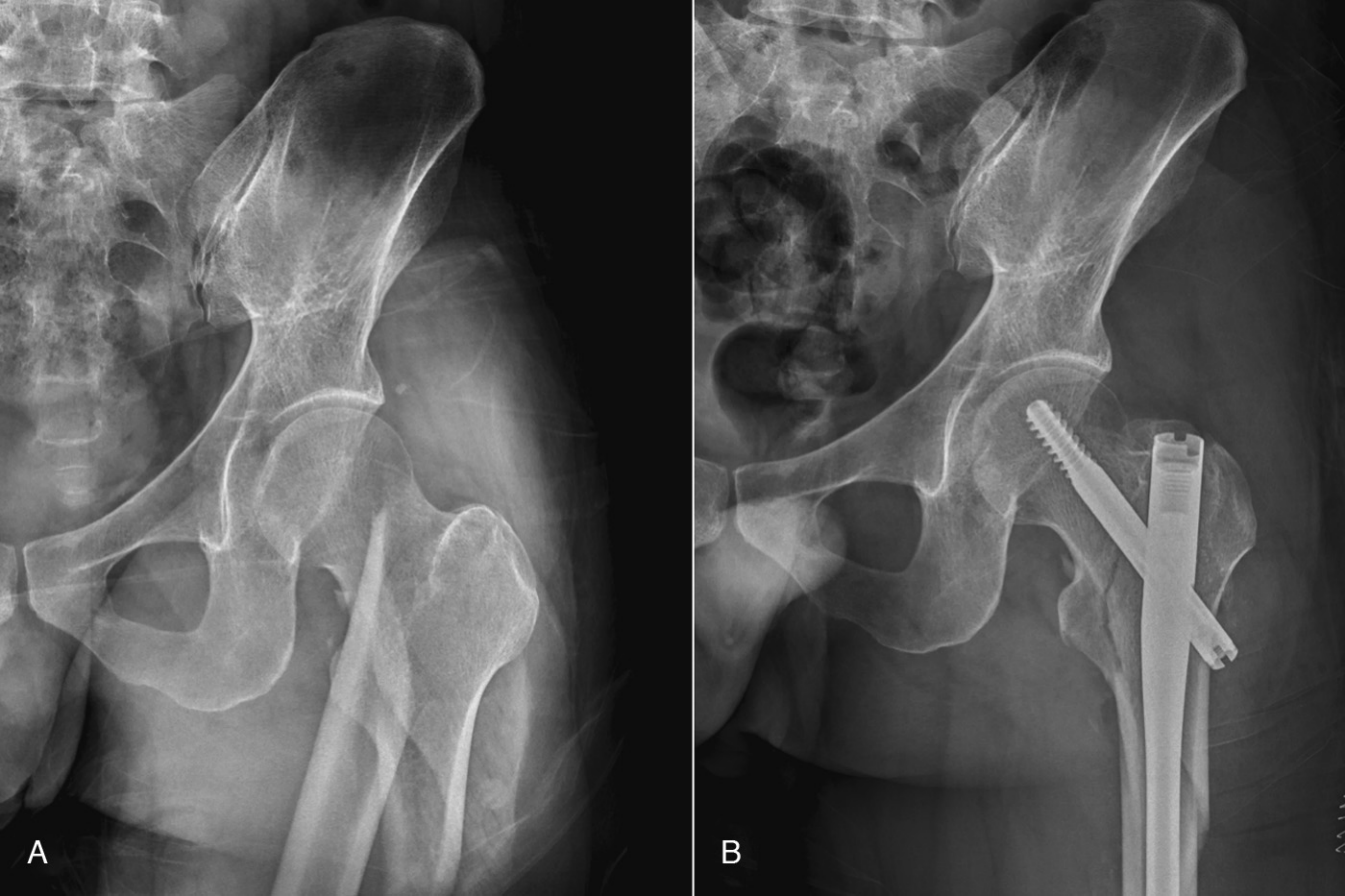
(A) Left femoral intertrochanteric fracture with subtrochanteric extension, and (B) the status after open reduction and internal fixation with an intramedullary nail.

A 35-year-old man without any underlying diseases suffered from a left femoral subtrochanteric fracture owing to a traffic accident (Fig. 1A). The patient's vital signs were stable. Open reduction and internal fixation with an intramedullary nail were performed the next day, specifically 21 hours after the traffic accident (Fig. 1B). Nine hours after the surgery, drowsiness and blurring of vision occurred in the right eye, which was described as focal darkness. Dyspnea emerged approximately 29 hours postoperatively. His vital signs were as follows: tachycardia (115 beats/min), tachypnea (24 breaths/minute), 84% hemoglobin saturation with ambient air, and blood pressure of 106/62 mmHg. His Glasgow Coma Scale score was E3V3M5. Upon neurological examination, left homonymous hemianopia was detected through confrontation test. Ophthalmic examination showed unremarkable anterior and posterior segments with no enlarged optic disc cupping in both eyes; therefore, occipital lobe infarction-related visual field defect was considered.

**Figure 2:**
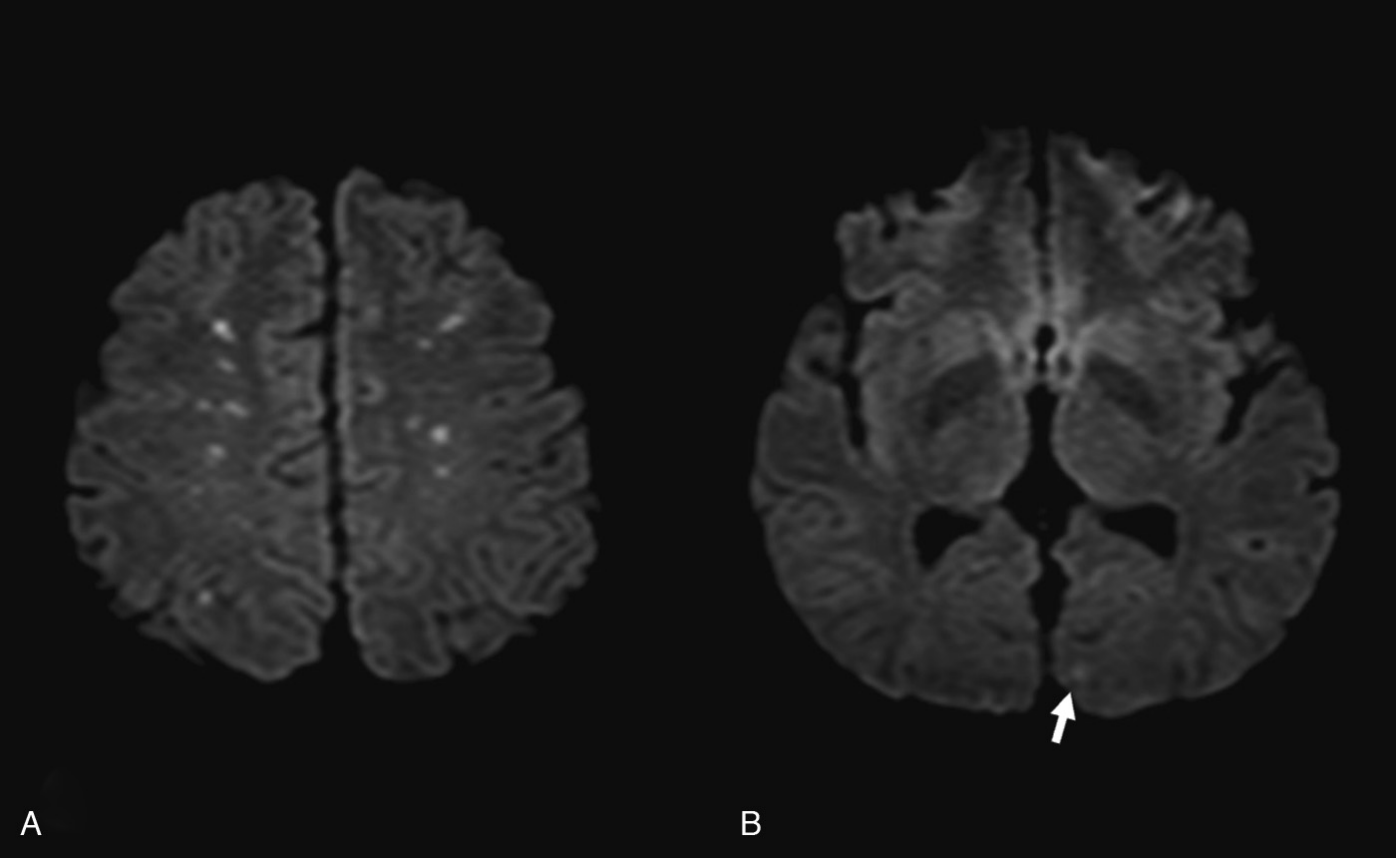
Diffusion weighted imaging series of brain magnetic resonance imaging (MRI) showed multiple disseminated small infarctions in the white matter of the (A) bilateral cerebral hemispheres and (B) left occipital region (arrow).

**Figure 3:**
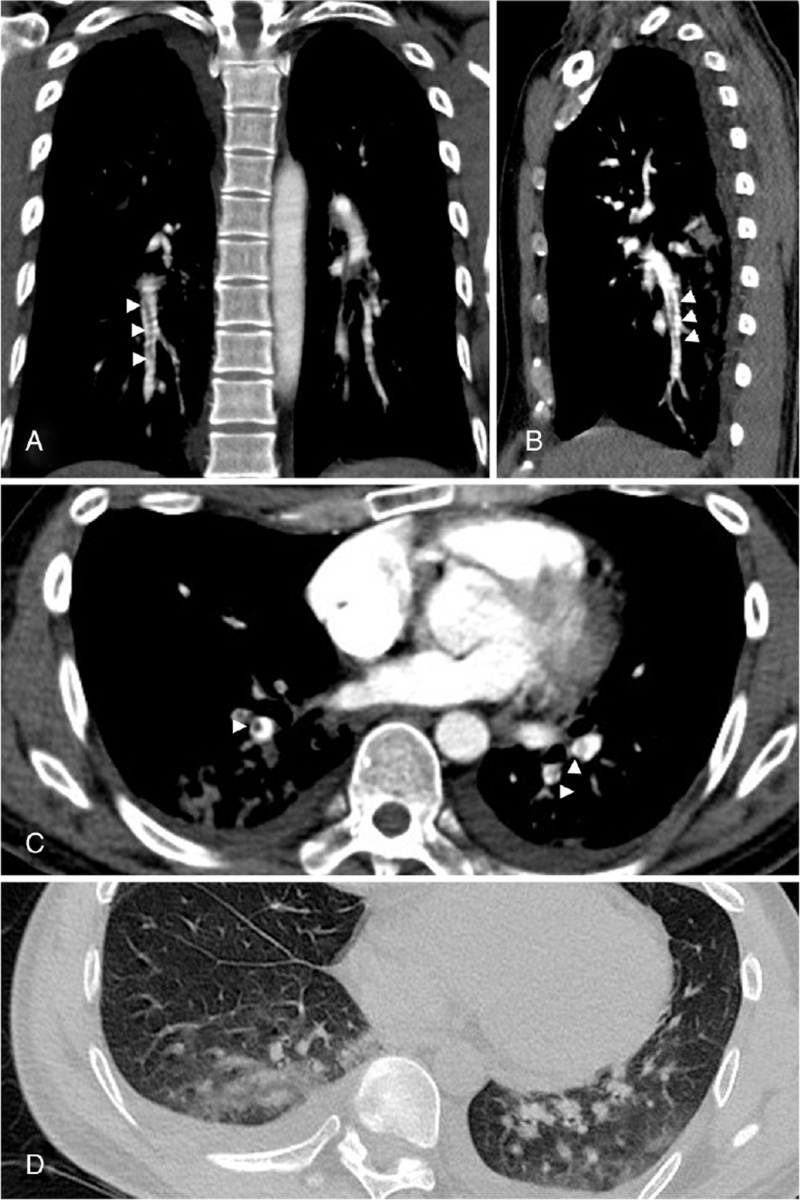
Computed tomography pulmonary angiography showed lower pulmonary artery embolisms (arrowheads) (A) coronal view (B) sagittal view (C) axial view and (D) lung window showed ground-glass opacities in bilateral lung.

Laboratory tests for early-onset stroke, including autoimmune profiles, antiphospholipid antibodies, protein C and protein S, lipid profiles, and glycosylated hemoglobin, were within normal limits (Table 1). The level of hemoglobin decreased significantly from 13.5 g/dL (Day 2) to 9.7 g/dL (Day 3), and the platelet count showed reactive thrombocytosis immediately after pulmonary embolism and dropped from 317,000/mm^3^ (Day 2) to 139,000/mm^3^ (Day 3). D-dimer was 8.53 mg/L on day 3. Brain magnetic resonance imaging (MRI) showed multiple disseminated small infarctions in the white matter of both cerebral hemispheres, including the left occipital lobe in the diffusion-weighted imaging series (Fig. 2). Computed tomography pulmonary angiography (CTPA) revealed lower pulmonary artery embolism, and the density of emboli was mixed up with fatty density component and thrombotic density component. Ground-glass opacities in bilateral lung were noticed as well (Fig. 3). Cardiac echocardiography showed no intracardiac shunts, such as the patent foramen ovale or significant right ventricular dysfunction signs after TPE. Two-point compression ultrasonography for lower extremity showed no deep venous thrombosis. No atrial fibrillation was observed on the Holter scan.

**Table 1:** Characteristic of the serum hematologic profile, biochemistry, vascular risk factors, and autoimmune profiles.

	OP Day		Post-OP Day 1	
WBC	11150	/μL	11990	/μL
RBC	511 × 10^4^	/μL	330 × 10^4^	/μL
Hemoglobin	15.1	g/dL	9.7	g/dL
Platelet	39 × 10^4^	/μL	13.9 × 10^4^	/μL
AST	22	U/L	45	U/L
ALT	24	U/L	32	U/L
BUN	11.6	mg/dL	10.2	mg/dL
Creatinine	0.9	mg/dL	0.55	mg/dL
D-dimer	–		13.5	g/dL

Vascular risk factors			Autoimmune profiles		
CHOL	202	mg/dL	ANA	<1:40	
HDL	55.2	mg/dL	aPL-IgG	<5.1	GPL
LDL	145	mg/dL	aPL-IgM	24.5	MPL
Hb A1C	5.3	%	aCL-IgG	0.6	GPL-U/mL
Homocysteine	8.18	μmole/L	aCL-IgM	1.6	MPL-U/mL

Hematologic profiles					
Antithrombin III	98	%	Protein C	91	%
Clotting Factor VIII	352	%	Protein S	116	%

aCL = anti-cardiolipin, ALT = alanine aminotransferase, ANA = antinuclear antibody, aPL = Anti-phospholipid, AST = aspartate aminotransferase, BUN = blood urea nitrogen, CHOL = total cholesterol, Hb A1C = glycosylated hemoglobin, HDL = high-density lipoprotein, LDL = low-density lipoprotein, OP = operation, RBC = red blood cell, WBC = white blood cell.

For treatment, initially heparin was administered as a bolus dose of 80 U/kg and maintained 18 U/kg/hr for one day, and the next day the anticoagulant medication was shifted to 10 mg apixaban, twice daily, for a week. The dose of apixaban was tapered to 5 mg twice daily later. Both consciousness and oxygenation improved quickly on Day 6 without major bleeding events. The level of serum D-dimer decreased to 2.64 mg/L on Day 19.

**Figure 4:**
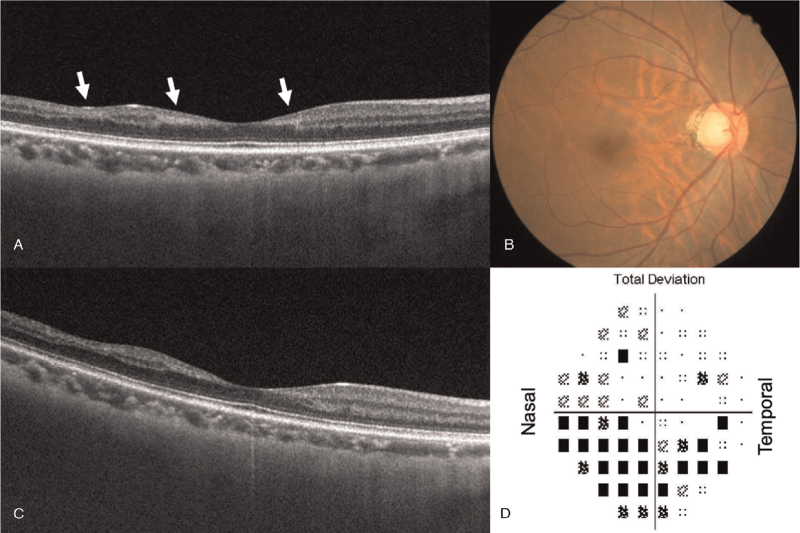
Paracentral acute middle maculopathy associated with fat embolism syndrome. (A) The horizontal cross-sectional image at 3 months postoperatively shows hyperreflectivity in the middle retinal layers (arrows) indicating focal deep capillary ischemia. At 6 months postoperatively, (B) an enlarged optic disc cupping can be observed on the color fundus photograph, and (C) progressive thinning of the temporal macula can be seen on the OCT horizontal slab. (D) The automated static perimetry demonstrates scotomas especially in the inferior-nasal quadrant.

At the third month examination, the visual field defects persisted in the right eye, and a thorough ophthalmic examination showed enlarged optic disc cupping in the right eye; however, thinning of the peripapillary retinal nerve fiber layer was absent on optical coherence tomography (OCT). Instead, paracentral acute middle maculopathy with thinning of the temporal macula was noted on OCT and corresponded to the scotomas on automatic static perimetry. After six months, progressive thinning of the temporal macula and persistence of scotomas on perimetry were observed (Fig. 4). This indicated that the visual field defects were caused by focal ischemia of the inner retina, which was likely related to fat emboli in the deep retinal capillaries.^[6]^ At the sixth month, the follow-up level of D-dimer was within normal limits, and we discontinued using NOAC. Cardiovascular and cerebrovascular events were absent during the follow-up period.

## 3 Discussion

FES is a clinical diagnosis presenting with respiratory distress, neurological deficit, and petechial rash. There are two theories regarding its mechanical and biochemical mechanisms. Furthermore, the mechanical theory is classified into paradoxical embolism, which involves the patent foramen ovale (PFO), and microembolism, which could explain the development of FES in patients without PFO.^[7]^ Biochemical response involves the production of toxic intermediaries of circulating fat and supplements the mechanical theory.^[8]^ Both are based on fat leakage into the vessels, and can occur in patients with long bone fractures.

The surgical timing and technique can play a significant role in the outcome of FES. Patients who underwent intramedullary nailing within 10 hours after injury were at a lower risk of developing FES.^[9]^ A meta-analysis of small randomized controlled trials showed that prophylactic corticosteroids would reduce the risk of FES by 78%.^[2]^ However, there are no evidence-based recommendations for the prevention of FES,^[10]^ and its incidence due to long bone fractures has decreased over the last few decades.^[10]^

In our case, the diagnosis of FES was made using Gurd's criteria^[11]^ (two majors: neurologic deficit and respiratory distress; two minors: tachycardia and sudden thrombocytopenia) and Schonfeld's criteria^[12]^ (diffuse alveolar infiltrates and hypoxemia). Brain MRI revealed disseminated hyperintense lesions in the DWI series, which also supported the diagnosis. Meanwhile, a recognizable filling defect of the pulmonary arteries bilaterally on computed tomography angiography and elevated D-dimer levels established the diagnosis of PE.

Well's criteria^[13]^ is the first-line screening for diagnosing TPE. However, in the acute setting of a patient with femoral fracture, it is difficult to distinguish whether respiratory distress is secondary to FES or TPE after excluding evident cardiogenic and pulmonary illnesses. In Well's criteria, the absence of an alternative diagnosis of TPE (3 points) is usually observed. This indicated a high probability of exceeding 4 points, which is considered as the cut-off value to exclude TPE. In our case, the patient underwent the operative procedure with a heart rate of 100 beats/minute, which indicated a Well's score of 6 (moderate risk). Generally, patients with post-traumatic FES that require surgery, such as intramedullary nailing or prolonged immobilization, are usually at moderate or high risk based on the Well's criteria. Therefore, CTPA was strongly recommended.

Anticoagulants, such as heparin, can stimulate lipase activity and increase the clearance of lipids from the circulation. However, free fatty acids are increased and exacerbate the underlying proinflammatory reaction.^[14]^ Despite insufficient evidence to support its routine use in FES, cases of fulminant FES and low risk of bleeding, such as in the present case, showed quicker recovery and good prognosis without major bleeding, such as internal or severe wound bleeding. Ebina et al.^[15]^ reported the first case of hip fracture with concomitant FES and PE, wherein heparin infusion was indicated based on PE. We initially administered heparin and switched to apixaban afterwards. To the best of our knowledge, the case described in this study is the second case of hip fracture with concomitant FES and TPE and the first to use NOACs. Another study also showed that NOACs are the preferred treatment in acute PE.^[16]^ Among NOACs, apixaban showed the most favorable safety profile a with statistically reduced risk of major bleeding events.^[17]^ Due to avoidance of routine coagulation monitoring and reduced bleeding, it provided greater convenience for clinicians and patients.^[18]^

In conclusion, it is difficult to differentiate FES from TPE in patients with hip fracture and respiratory distress, especially if these occur concomitantly. The current diagnostic criteria are confined to clinical similarities and common predisposing factors. Furthermore, aggressive diagnosis of PE with CTPA is necessary so that the anticoagulant therapy can be started early instead of supportive care for FES.

## Acknowledgments

This research did not receive any specific grant from funding agencies in the public, commercial, or not-for-profit sectors. All contributors meet the criteria for authorship of International Committee of Medical Journal Editors.
